# Fabrication of all-transparent polymer-based and encapsulated nanofluidic devices using nano-indentation lithography

**DOI:** 10.1038/micronano.2016.84

**Published:** 2017-03-27

**Authors:** Cong Wu, Tiffany G. Lin, Zhikun Zhan, Yi Li, Steve C.H. Tung, William C. Tang, Wen J. Li

**Affiliations:** 1 Department of Mechanical and Biomedical Engineering, City University of Hong Kong, Hong Kong 999077, China; 2 Department of Biomedical Engineering, University of California, Irvine, CA 92697-2715, USA; 3 Department of Automation, Yanshan University, Qinghuangdao 066004, China; 4 Department of Mechanical Engineering, University of Arkansas, Fayetteville, AR 72701, USA

**Keywords:** indentation lithography, nanochannel, nanofluidic device, nanofluidic flow

## Abstract

In this paper, we describe a novel and simple process for the fabrication of all-transparent and encapsulated polymeric nanofluidic devices using nano-indentation lithography. First, a nanomechanical probe is used to ‘scratch’ nanoscale channels on polymethylmethacrylate (PMMA) substrates with sufficiently high hardness. Next, polydimethylsiloxane (PDMS) is used twice to duplicate the nanochannels onto PDMS substrates from the ‘nano-scratched’ PMMA substrates. A number of experiments are conducted to explore the relationships between the nano-indentation parameters and the nanochannel dimensions and to control the aspect ratio of the fabricated nanochannels. In addition, traditional photolithography combined with soft lithography is employed to fabricate microchannels on another PDMS ‘cap’ substrate. After manually aligning the substrates, all uncovered channels on two separate PDMS substrates are bonded to achieve a sealed and transparent nanofluidic device, which makes the dimensional transition from microscale to nanoscale feasible. The smallest dimensions of the achievable nanochannels that we have demonstrated thus far are of ~20 nm depth and ~800 nm width, with lengths extendable beyond 100 μm. Fluid flow experiments are performed to verify the reliability of the device. Two types of colloidal solution are used to visualize the fluid flow through the nanochannels, that is, ethanol is mixed with gold colloid or fluorescent dye (fluorescein isothiocyanate), and the flow rate and filling time of liquid in the nanochannels are estimated based on time-lapsed image data. The simplicity of the fabrication process, bio-compatibility of the polymer substrates, and optical transparency of the nanochannels for flow visualization are key characteristics of this approach that will be very useful for nanofluidic and biomolecular research applications in the future.

## Introduction

The field of nanofluidics is widely known as the research and application of the behaviors of liquid flow in a specific area that is confined to the nanoscale^1^. A significant research interest has risen in this field due to the unique nanofluidic phenomena and flow properties that are markedly different from those in the well-developed microfluidics field. For example, since the dimensions of typical nanochannels are comparable to those of biomolecules, such as proteins and DNAs, they may be used for the transportation of selective molecules and DNA stretching. Furthermore, similar to microchannels, the surface-to-volume ratio of nanochannel structures is ultra-high, which leads to a diffusion-limited reaction^2^ and negative water pressure induced by capillarity^3^. Moreover, because the size of the electrical double layer formed by strong electrostatic interactions between ions and the charged surface is in the range of 1−100 nm (Refs. 4,5), nano-flows in nanochannels will have flow properties that significantly deviate from Newtonian fluids. Based on these novel dimensional effects, many significant applications have been explored over the past 10 years in both basic research and applied studies (especially in the fields of biology, chemistry, and medicine), ranging from the control of molecular transport and analysis of DNA or other single molecules to energy conversion, sample separation systems, and nanofluidic electronics^6–10^. For example, artificial nanochannels act as an excellent biomimetic tool in biomolecular research^6,8,9^. In addition, extremely sensitive biosensors that consist of a single nanochannel have been explored for the detection and analysis of various biological molecules such as DNA, proteins, and ions^11^. For nanochannel arrays, selective transport for electrical signaling in nerves and muscles has recently been developed^12^. Thus, an ideal nanofluidic device has the critical requirement of having nanochannels with span-wise dimensions that are comparable to molecules, that is, with width and depth dimensions spanning from sub-nanometers to a few nanometers.

Various techniques for fabricating nanofluidic devices have been explored over the past decade^13–15^. Traditionally, electron beam lithography^16^, focused ion beam lithography^17^, and nanoimprint lithography^10,18^ have mostly been used to create nanometer-sized structures in nanofluidic devices in the past. For the fabrication of the nanochannels, these conventional approaches have definite advantages with respect to a high level of controllability, resolution, and reproducibility. However, critical limitations remain, as these processes are typically complex, expensive, and time-consuming. Meanwhile, an increasing number of unconventional fabrication techniques have also been developed in recent years^19^. For example, some polymers, such as polycarbonate^20,21^ and polymethylmethacrylate (PMMA)^22^, have desirable material properties, whereby they can decompose and form nanoscale structures on the surfaces when they are exposed to UV light or treated in a thermal compression process^23^. Although these methods provide alternative solutions for nanofabrication that are typically simple and low-cost, they show limitations in terms of the fabrication success rate and throughput, and they have insufficient control of the geometric dimensions for the fabricated nanochannels. Therefore, a technique that comprehensively meets the technical demands of nanofluidic devices, with significant cost reduction, is urgently required by nanofluidic researchers.

Considering the optimization of fabrication methods for precise dimensional control and cost-effectiveness, scanning-probe lithography, which mainly refers to the use of the scanning tunneling microscope and atomic force microscope (AFM)^24–30^ as ‘lithographic’ tools, is an emerging area in the field of nanofabrication. For example, the diamond tip of a cantilever installed in an AFM is used to mechanically pattern nanoscale features on the surfaces of substrates, such as silicon and metals. However, the depth of the structures fabricated using AFM changes from a few to tens of nanometers, which limits the technique and its application in fabrication at a larger scale. Similar to the principle of AFM-based nanomachining, the process of indentation lithography (IndL) via a nanoindenter was proposed in 2010 (Ref. 31) and has shown a remarkable potential for nanochannel fabrication. With a greater loading force applied to a probe tip, that is, the force can be varied from 100 nN to 1 N, it can scratch nanochannels on both hard and soft materials by a nanoindenter, and the resulting dimensions of the nanochannels have greater ranges in terms of the depth and width. In this paper, by taking advantage of IndL, we present a new fabrication process that is capable of creating an all-transparent and polymer-based (that is, polydimethylsiloxane or PDMS) nanofluidic device with nanochannel arrays, which have controllable channel sizes. The smallest dimensions of the achievable nanochannels that we have demonstrated thus far are ~20 nm depth and ~800 nm width, with lengths extendable beyond 100 μm. Liquid flow experiments are also conducted to prove the reliability of the device and observe the nanofluidic flow phenomenon. The details of the fabrication procedure and the related experimental results, including the estimations of the fluid flow rate in the nanochannels, are presented in this paper. The simplicity of the fabrication process, bio-compatibility of the polymer substrates, and optical transparency of the nanochannels for flow visualization are key characteristics of this approach that will be very useful for nanofluidic and biomolecular research applications in the future.

## Materials and methods

### Nanochannel fabrication

#### Parametric experiments

**Figure 1:**
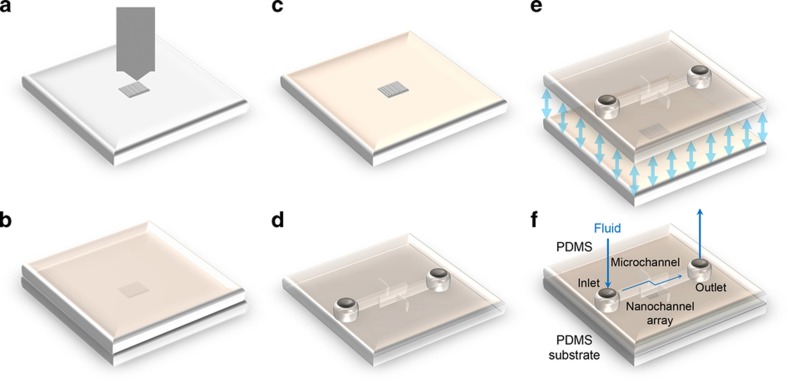
Schematic of the fabrication process for the nanofluidic device. (**a**) Nanochannel fabrication on a polymethylmethacrylate substrate by nanomechanical probes (the gray area in the center of the substrate represents the fabricated nanochannels). (**b**) Duplication of polydimethylsiloxane (PDMS) (twice). (**c**) The PDMS substrate with concave nanochannels. (**d**) Microchannel fabrication on a PDMS ‘cap’ substrate. (**e**) Alignment and bonding between the two PDMS pieces. (**f**) Sealed nanofluidic system.

**Figure 2:**
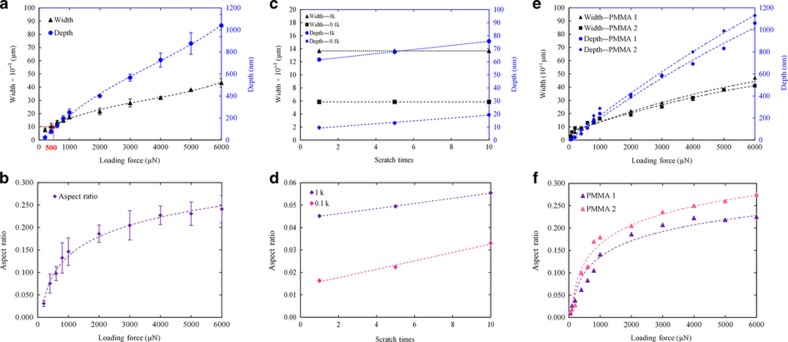
Width, depth, and aspect ratio of nanochannels fabricated using a conical-shaped probe. (**a** and **b**) Under a loading force varied from 200 to 6000 μN, with error bars showing the related standard deviations. (**c** and **d**) Under loading forces of 100 and 1000 μN, respectively, for *in situ* scratching performed once, 5 times, and 10 times. (**e** and **f**) Under a loading force ranging from 50 to 6000 μN for two different polymethylmethacrylate (PMMA) substrates.

An overview of the IndL-based nanochannel device fabrication process is shown in Figure 1. A nanoindenter (TI 700 Ubi 1, Hysitron, Inc., Minneapolis, MN, USA) equipped with a conical-shaped indenter (a form of cono-spherical probe with a radius of 1 μm) is used to scratch the surfaces of PMMA substrates with a constant loading force that is controlled by the corresponding software (TriboScan version 8, Hysitron Incorporated, 10025 Valley View Rd., Minneapolis, MN 55344, USA) for nanochannel fabrication. During scratching, the indenter is first pressed into the surfaces of PMMA substrates. The indenter tip then performs a rectilinear movement with a constant loading force, and then the tip is vertically withdrawn from the surface after scratching. Thus, the formation of the nanochannels mainly depends on the substrate deformation, that is, the materials under the probe pile up on both sides and at the end of a channel during scratching. In addition, a small amount of loose PMMA residues are left in the channel, and they can easily be removed by ultrasonic DI water cleaning. A series of preliminary experiments are conducted to explore the various parameters that govern nanochannel production via this method. For a single scratch, the nanochannel length is 10 μm and the fabrication duration is ~5 min. Moreover, nanochannels with different aspect ratios (the ‘aspect ratio’ here is defined as the depth of the channel divided by the width of the channel) are fabricated, as the applied loading force is varied. The results indicate that nanochannels fabricated this way are reproducible. For example, under a loading force varying from 200 to 6000 μN, nanochannels are repeatedly fabricated using a specific loading force three times. Figures 2a and b, respectively, show the average values of the channel width, depth, and aspect ratio based on the three sets of AFM (Agilent 5500, Agilent Technologies Inc., 1601m California Street, Palo Alto, CA 94304, USA) measurement results; the related standard deviations are also calculated and shown as error bars in the figures. It can be observed that the dimensions of the fabricated nanochannels are generally related to the magnitude of the loading forces as follows: the width, depth, and aspect ratio increase as the applied loading force increases. In addition to the applied loading force, other experimental conditions such as the operational temperature and scratching speed also have an influence on the dimensions of the fabricated nanochannels^32^. The related parameters are kept constant during each experiment, that is, the working temperature is room temperature (~22 °C), and the running speed of the probe is 0.33 μm s^−1^. Under all the above conditions, channels with nanoscale widths and depths are created when the loading force is below ~500 μN; currently, the smallest dimension of the achievable nanochannels is a depth of ~20 nm and a width of ~800 nm under an applied force of 200 μN. Thus, this approach exhibits flexibility in the fabrication of nanochannels with specific aspect ratios by applying the corresponding loading forces. Therefore, the nanoscratching method is an effective, reliable, and repeatable method for fabricating nanochannels with controllable aspect ratios on PMMA substrates, according to the intended application.

**Figure 3:**
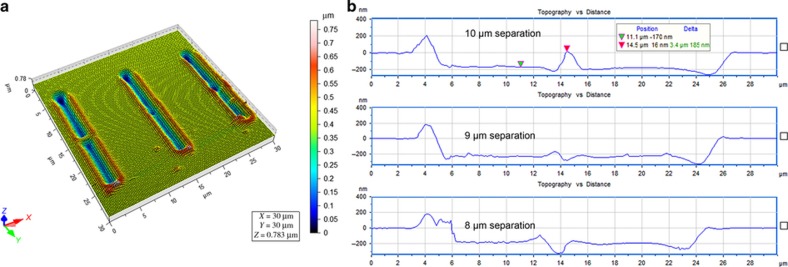
(**a**) Three-dimensional atomic force microscope image of three long nanochannels with different separation distances of 10, 9, and 8 μm between two contiguous scratches, respectively, from left to right. (**b**) The profiles that correspond to the three long nanochannels.

Deeper and longer nanochannels can be made on PMMA substrates by repeatedly scratching the surface in the length-wise direction. The ‘separation distance’ between each scratch leads to different effects. Here, the ‘separation distance’ is defined as the distance between the initial locations where the probe tip starts to scratch the successive 10-μm-long channels in the length-wise direction. Therefore, when the separation distance is equal to 0 μm, deeper nanochannels are fabricated, as the tip is made to repeatedly scratch the same channel. When the separation distance ranges between 0 and 10 μm, longer nanochannels are obtained; meanwhile, larger distances (for example, 11 μm) will result in disconnected nanochannels. Figures 2c and d show the dimensions of deeper nanochannels fabricated in this manner, highlighting that higher aspect ratios can be achieved by increasing the number of *in situ* scratches. To fabricate longer nanochannels, sequentially ‘connected scratches’ with an appropriate separation distance are critical. As shown in Figure 3a, for each long nanochannel, the two contiguous scratches have different separation distances, that is, 10, 9, and 8 μm, respectively, from left to right. As observed from the figure, there are residuals left in the joint points between each scratch when the separation distance is 10 μm, which leads to channel discontinuity and could have an influence on liquid flow in the nanochannels. When the separation distance decreases to 9 and 8 μm, the excess materials inside long nanochannels are completely removed, and this ensures the connectivity of the channels. Note that the nanochannel with the separation distance of 8 μm is shorter than the nanochannels fabricated with a separation distance of 9 μm, which means that choosing a separation distance of 9 μm is more efficient for the fabrication of longer nanochannels. In addition, Figure 3b shows the length-wise cross-section profiles of the three long nanochannels fabricated using separation distances of 10, 9, and 8 μm (10 μm for the top profile, 9 μm for the middle profile, and 8 μm for the bottom profile). Therefore, considering the channel continuity and fabrication efficiency, the separation distance of 9 μm is appropriate for fabricating long nanochannels. For example, a nanochannel with a length of 190 μm can be achieved by continuous scratching performed 21 times with a separation of 9 μm; the total required time is approximately 1.5 h.

In addition, the physical properties of PMMA substrates, such as the hardness and Young’s modulus, also play an important role in the resulting dimensions of the nanochannels fabricated via the scratching technique. Two PMMA substrates with slightly different properties are used to explore the influences of these properties. PMMA 1 has a hardness of 192.10 MPa and Young’s modulus of 4.10 GPa; for PMMA 2, the values, respectively, are 173.65 MPa and 3.96 GPa. Figures 2e and f show two sets of parameters (the width, depth, and aspect ratio) of nanochannels scratched on the two PMMA substrates. From the results, the substrate property does NOT have an obvious effect on the widths of the fabricated nanochannels. However, the substrate with a smaller hardness and Young’s modulus results in larger depths, which lead to larger aspect ratios of the nanochannels under the same loading force. Moreover, for the same types of material with different hardness values and Young’s moduli, the variation trends for the width, depth, and aspect ratio are similar to each other as the applied load is increased. Thus, the parameters for the physical properties of materials need to be considered during fabrication to create specific nanochannels.

**Figure 4:**
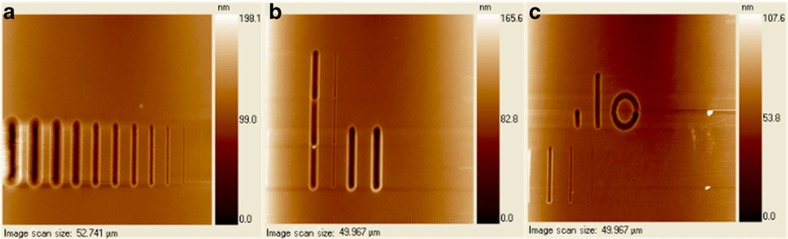
Nanoindenter scanned images. (**a**) Nanochannels fabricated by scratching once with a constant loading force varied from 50 to 5000 μN (from right to left). (**b**) Longer and deeper nanochannels fabricated by scratching many times in the length-wise direction with different loading forces; the two shorter channels on the right were scratched repeatedly with a ‘separation distance’ of 0 μm. (**c**) Straight and nonlinear nanochannels fabricated by indentation.

In addition to the scratching approach, the indentation approach, which is another major function of a nanoindenter, has also been explored for creating both straight and non-linear nanochannels on PMMA substrates. Nanochannels are fabricated by producing successive indentations with the probe; the process requires approximately 80 min to indent a straight nanochannel with a length of 10 μm and nearly 2 h to produce a circular channel with a radius of 5 μm. Although the aspect ratios of the nanochannels fabricated by the nanoindentation process are lower and the required fabrication time is longer than the corresponding results obtained from nanoscratching, indentation remains a promising method for fabricating complex-shaped nanochannels. Figure 4 shows the nanochannels fabricated by a conical-shaped tip under different working modes of a nanoindenter.

**Figure 5:**
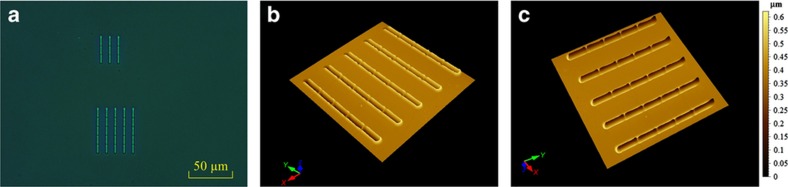
(**a**) Optical microscope image of two nanochannel arrays consisting of 3×3 scratches and 5×5 scratches, and three-dimensional atomic force microscope images of the 5×5 nanochannel array; (**b**) top view; and (**c**) bottom view.

Based on the technique of scratching by nanomechanical probes, nanochannel arrays are designed and fabricated on PMMA substrates, as the first step for the preparation of a nanofluidic device that involves the integration of multiple nanochannels and provides more opportunities for liquid flow in one device; thereby, the observation of the flow phenomenon in nanochannels is simplified and efficiency is improved. Loading forces of 1000 and 2000 μN are applied to scratch the nanochannel arrays on the PMMA substrates, which have a hardness of 192.10 MPa and Young’s modulus of 4.10 GPa; the operational temperature during the scratch process is maintained at room temperature (~22 °C). From the AFM measurements, the width and depth for one nanochannel fabricated under a loading force of 1000 μN are 1.7 μm and 240 nm, respectively; for a loading force of 2000 μN, the width and depth are 2.2 μm and 410 nm, respectively. Figure 5 shows the images of nanochannel arrays obtained by both an optical microscope (BX60F5, Olympus Optical Co., Ltd., Shinjuku, Tokyo, Japan) and an AFM.

#### Nanomolding

**Figure 6:**
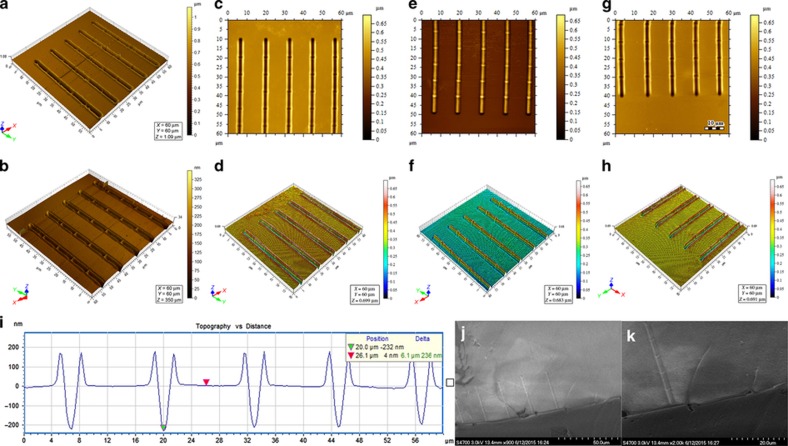
Two- and three-dimensional atomic force microscope images of nanochannel arrays. (**a** and **b**) Nanochannels on the same polymethylmethacrylate (PMMA) substrate before and after molding, respectively, at a post-baking temperature between 75 and 80 °C. (**c** and **d**) Concave nanochannels scratched on a PMMA substrate. (**e** and **f**) Convex nanochannels on a polydimethylsiloxane (PDMS) substrate after molding for the first time. (**g** and **h**) Concave nanochannels on another PDMS substrate after molding twice. (**i**–**k**) Cross-sectional profile and scanning electron microscope images of the nanochannel array on the PDMS substrate.

Using the PMMA substrates with the nanochannel arrays as molds, the process of transferring the nanoscale structures from PMMA to PDMS is another critical nanofabrication step because PDMS has notable advantages for molding and sealing compared with other types of polymers. First, the nanochannels on PMMA substrates are replicated through nanomolding, resulting in *convex* nanochannels on PDMS substrates. Second, the PDMS substrates with *convex* nanochannels are coated with a thin silane layer for anti-adhesion, and the amount of time required for silylanization is approximately 4 h. Third, the nanochannels are transferred by nanomolding for the second time from the silane-treated PDMS substrates to the new PDMS substrates to create the corresponding *concave* nanochannels. During the fabrication process, PDMS (Sylgard 184 SIlicone Elastomer, Dow Corning Corporation, 2200 W. Salzburg Rd., Auburn, MI 48611, USA), including a base and curing agent, is used; in addition, the mix ratio by weight between the two components is 10:1. Optimization experiments for determining the appropriate curing conditions are performed, and the results demonstrate that the post-baking protocol at 70 °C for 2 h maintains both the geometry and dimensions of the original PMMA nanochannels relatively well after molding is performed, that is, the dimensional tolerances are approximately 5–10%. However, a higher temperature for PDMS curing leads to serious deformation of the PMMA substrates (the range of the glass transition temperature is generally from 85 to 165 °C for PMMA), which ultimately results in changes to the nanochannel dimensions on the PDMS replicas. For example, if the width and depth of the original scratched nanochannels on PMMA are, respectively, 1.5 μm and 245 nm, after molding is performed twice at a curing temperature of 80 °C, the width of the duplicated PDMS nanochannels changes to 1.1 μm, and the depth decreases to 48 nm. Figures 6a–h show a series of scanned images of nanochannel arrays using an AFM, including a comparison of the nanochannel shapes on a PMMA substrate, which deforms after molding at a temperature higher than 70 °C. Both the two- and three-dimensional images of a nanochannel array scratched on another PMMA substrate and then molded by PDMS twice using the optimized protocol described in this paper are also shown. Figures 6i–k show a cross-sectional profile and scanning electron microscope images of the nanochannel array on the PDMS substrate. In addition, the surface roughness (*R*_a_) of the concave nanochannels fabricated on the PDMS substrate is examined by an AFM since the surface properties, such as roughness, play an important role in nanofluidic devices for applications in the related biomolecular research field. For example, the roughness influences the interactions between biomolecules and surfaces, such as cellular adhesion and protein adsorption^33,34^. From the results, *R*_a_ of the internal wall of the nanochannels on PDMS is approximately 10±2 nm when the loading force used for scratching the channels is 1000 μN, and the value increases to 20±5 nm when the loading force changes to 2000 μN. Hence, a larger scratching force leads to a rougher surface.

### Microchannel fabrication

The fabrication of microchannels on PDMS ‘cap’ substrates is essential for producing encapsulated nanofluidic devices because microchannels play an important role in guiding liquid into the nanochannels and enable the transition of dimensions from the microscale to the nanoscale. Owing to the simplicity, repeatability, and reliability of conventional photolithography, this technique is used to generate the master with the microchannel patterns^35^. Then, soft lithography using PDMS is conducted to transfer the microchannels from the PMMA master to the PDMS ‘cap’ substrates.

**Figure 7:**
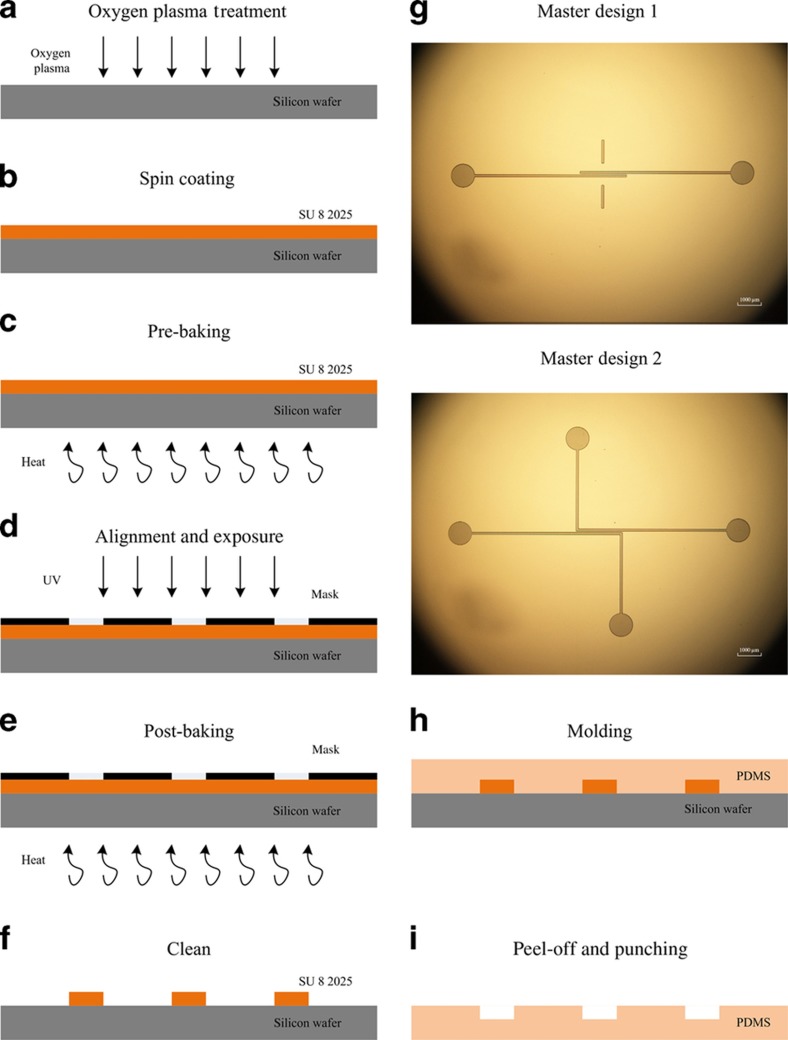
Schematic of the procedure for microchannel fabrication. (**a**) A silicon wafer is treated by oxygen plasma for 1 min. (**b**) A photoresist layer of SU8 2025 is spin-coated with a rotating speed of 4000 r.p.s. for 1 min. (**c**) Pre-baking is performed at 65 °C for 2 min and then at 95 °C for 5 min. (**d**) Mask alignment and ultraviolet (UV) exposure are conducted for 6 min using a UV aligner (Model 2000 Mask Aligner, OAI, 685 River Oaks Parkway, San Jose, CA 95104, USA). (**e**) Post-baking is performed at 65 °C for 1 min and then at 95 °C for 5 min. (**f**) The unexposed photoresist is cleaned using the developer. (**g**) Images of the two designed microchannel patterns on masters are obtained using an optical microscope. (**h**) Molding in polydimethylsiloxane (PDMS) is performed with a curing temperature of 70 °C for 2 h. (**i**) The cured PDMS is peeled off from the masters, and holes are punched for the inlet and outlet.

The following two opposing microchannel patterns are designed: one with two parallel microchannels that overlap 2 mm at the ends of each channel and the other with two overlapped parallel microchannels and an extended outlet at each bottom; the width of the microchannels is 100 and 200 μm, and the gap between each pair of overlapping microchannels is 50 μm. In addition, circles with a radius of 500 μm are located at the ends of the microchannels, as inlets and outlets. Figure 7 shows the procedure for microchannel fabrication, including the manufacture of masters on silicon wafers and the transfer of microstructures on the PDMS ‘cap’ substrates based on the two types of patterns above.

### Alignment and bonding

After the fabrication of one PDMS substrate with a nanochannel array and another PDMS ‘cap’ substrate with a pair of microchannels, the precise alignment and bonding are critical for sealing the open micro- and nanochannels as follows: the nanochannel array is aligned by hand under a microscope to cross the gap between the pair of microchannels to perform the connection, and the bonding through self-adhesion plays an important role in forming the encapsulated nanofluidic device. Compared to the oxygen plasma-bonding technique, which is widely used for permanent bonding, self-adhesion via van der Waals bonds between two surfaces of the PDMS substrates is reversible. Furthermore, the strength of its bond maintains the dimensions of the micro- and nanostructures while preventing leakage of the injected liquid. Bonding in this manner solves the problem of misalignment and increases the experimental efficiency for fluid flow due to the reusability of the device.

## Results

### Fluid flow experiment results

#### Experimental setup

A series of fluid flow experiments are conducted to verify the effectiveness of the polymer-based device and visualize fluid flow through the nanochannels. The system for these experiments consists of the following three parts: liquid injection, a nanofluidic device, and a vacuum supply. Based on the capillarity, which describes the phenomenon where a liquid flows in narrow spaces only by intermolecular forces between the liquid and its surrounding solid surfaces, the solution is first injected into the inlet of the nanofluidic device through a syringe; next, the liquid should flow, without the application of any pushing or sucking force, along the microchannel connected to the inlet, cross the intermediate nanochannel array where the phenomenon of nanofluidics occurs, and then reach the outlet microchannel. The outlet is connected to a set of vacuum systems, which involves a pump and glass bottle for providing continuous suction to the device when necessary in case the liquid is blocked within the channels.

#### Non-fluorescent experiments

**Figure 8:**
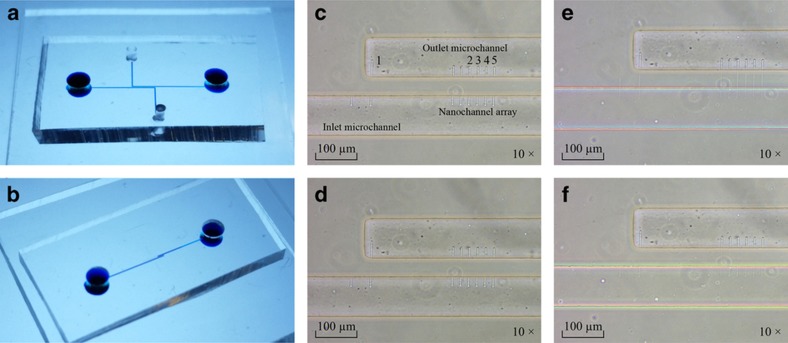
Optical microscope images of liquid flow experiments. (**a** and **b**) The devices are filled with a blue dye without leakage. (**c**) The sealed nanofluidic channels without a liquid. (**d**) The sealed nanofluidic channels after injection of the gold colloid mixed with pure ethanol, and the liquid begins to flow into the nanochannels. (**e**) Gradually, most nanochannels are filled with liquid. (**f**) After a few additional minutes, the visible nanochannels disappear again.

Due to the transparency of the nanofluidic device, a blue dye (McCormick & Company Inc., 18 Loveton Circle, Sparks, MD 21152, USA) is first injected into the systems through the inlet and the outlet on the PDMS ‘cap’ substrates with the two types of microchannel designs to ensure that the device is adequately sealed. Figures 8a and b illustrate that no leakage occurs in all of the devices; however, the solution cannot be observed through those nanochannels because the particle size is between 1 and 5 μm, which is larger than the dimensions of the nanochannels. Second, a gold colloid (with a particle size of 50 nm) mixed with pure ethanol (absolute ≥99.8%, Sigma-Aldrich Co., 3050 Spruce Street, St. Louis, MO 63103, USA) is selected as the injection liquid into the device with the PDMS ‘cap’ substrate of design 1, and the dilute solution appears nearly transparent instead of as a red color. After injection, the flow of liquid is observed through the movement of the air-liquid interface in the microchannels; however, it is difficult to view the liquid flow in nanochannels directly due to the diffraction limit. The possible appearance of flow relies on the change in the optical path by the liquid under the microscope, which enables a visual difference to be used to verify that the liquid flows across the nanochannels.

**Figure 9:**
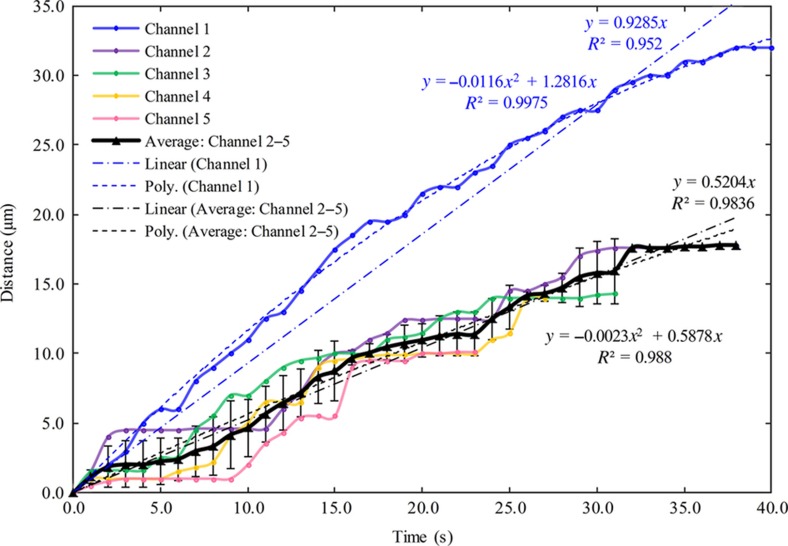
Scatter chart of flow distances per second for the liquid through nanochannels 1–5 in the polymer-based and encapsulated nanofluidic device.

As shown in Figures 8c–f, the part of the nanochannels under the PDMS ‘cap’ substrate cannot be easily observed under a microscope before liquid injection; in contrast, these nanochannels become visible after liquid injection. After a few minutes, the nanochannels gradually disappear again due to the liquid evaporation and absorption by PDMS. Based on the video for the liquid flow process in nanochannels, the flow distances per second through randomly selected nanochannels, marked as 1−5 in Figure 8c, are captured, as shown in Figure 9. Nanochannel 1 is fabricated with a loading force of 2000 μN, and nanochannels 2–5 have the same dimensions that are created under a loading force of 1000 μN.

From the experimental data, a linear function and a second-order polynomial function are used to fit curves of the flow distance in nanochannel 1 and the average flow distance in nanochannels 2–5, respectively. To simplify the computation, the linear regression results for the flow distance and time relationship are adopted to estimate the flow rates in the nanochannels. Thus, the liquid velocity of flow in nanochannel 1 is approximately equal to 0.93 μm s^−1^, and it is approximately 0.52 μm s^−1^ in nanochannels 2–5. Assuming that the cross-sectional shape is similar to a triangle for a nanochannel and a rectangle for a microchannel, then, the volume flow rates in the nanochannels are calculated as 0.419 and 0.106 μm^3^ s^−1^, respectively, for nanochannel 1 and nanochannels 2–5. Furthermore, the volume for an inlet/outlet microchannel with a length of 6.5×103 μm, a width of 100 μm, and a height of 10 μm is calculated as 6.5×106 μm^3^. Hence, we estimate the time it takes for the fluid to fill the outlet microchannel through nanochannel 1 as 183 days and that through nanochannels 2–5 as 708 days. We note that the estimation of the liquid-filling time through the nanochannels established above is valid only under the condition of the liquid being a mixture of gold colloid and pure ethanol, as used in our experiments, since different liquids or colloidal solutions will have different properties that may affect the flow phenomenon.

We note here that the capillary filling of nanochannels has been investigated by many researchers in the past, and, generally, the filling law of a pure liquid follows the Lucas-Washburn equation, which describes a relationship between the flow distance (*S*) and the square root of time (*t*)^36,37^. In our research, a mixture of gold colloid and pure ethanol is used for the liquid flow experiment and estimation of flow rate in the nanochannels. Since the colloidal solution used in our experiments is more complex than pure liquid, the filling law for the relationship between *S* and *t* may deviate from the Lucas-Washburn equation, as discussed in Ref. 38. More fluid flow experiments will be performed in future to explore the relationship between the flow distance and time in the nanofluidic device.

#### Fluorescent experiments

**Figure 10:**
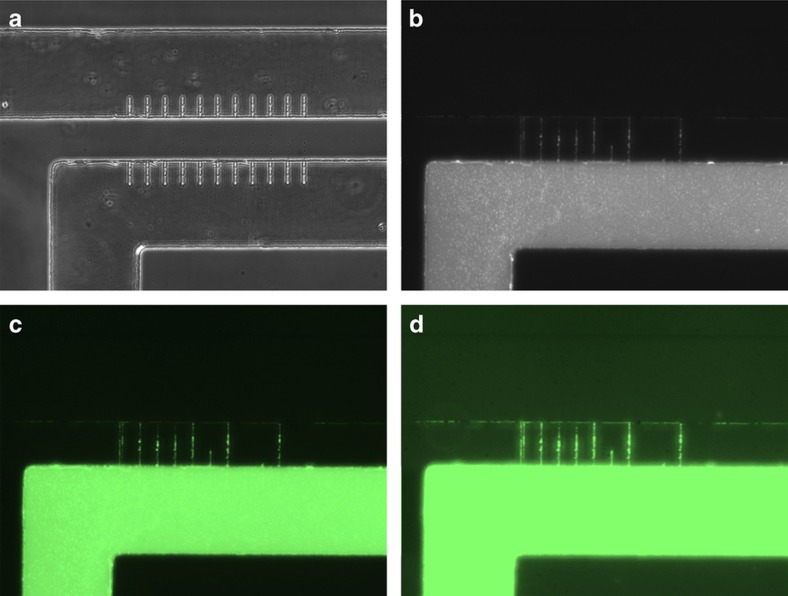
Optical microscope images of the liquid flow experiment with fluorescein isothiocyanate (FITC). (**a**) The sealed nanofluidic channels without a liquid. (**b**) The sealed nanofluidic channels after FITC solution injection with an exposure time of 5 s. (**c** and **d**) The processed images of the sealed nanofluidic channels after FITC solution injection with exposure times of 5 and 10 s, respectively.

Fluorescein isothiocyanate (FITC), which is a fluorescent dye (with an excitation wavelength of 495 nm and an emission wavelength of 519 nm) that is commonly used in nanotechnology, is a type of chemical compound that fluoresces when exposed to a particular wavelength of light. FITC provides the definite advantage of flowing without particle size problems, and it can be observed in the nanochannels of a transparent device. Moreover, the fluorescent dye is more effective for detecting liquid leakage compared with other colorless solutions. Thus, a FITC solution mixed with pure ethanol is injected into the device with the PDMS ‘cap’ substrate of design 2 and the PDMS substrate with a nanochannel array, which consists of the same 11 nanochannels with the width of 2.2 μm and depth of 410 nm fabricated under the loading force of 2000 μN. Figure 10 shows the results of the fluorescent experiment, which demonstrates that there is no liquid leakage in the device and the nanofluidic phenomenon occurs within the system.

## Discussion

The entire fabrication process for the nanofluidic devices presented in this paper is reliable and repeatable; however, the alignment between nanochannels and microchannels remains a problem. The top surface of the PDMS substrate is first focused, and the nanochannel array is moved to the center of the field of vision; the substrate is then located under the microscope, and the PDMS ‘cap’ substrate is then held by hand and moved left and right for alignment above the bottom substrate. When the upper layer is moved to the position where the nanochannels cross the microchannels, it is placed down vertically for bonding. The manual step takes a large amount of time and may easily lead to misalignment. Moreover, the misalignment will influence the fluid flow in the channels.

Another challenge of this work is the observation of fluorescent flow in the nanochannels. The amount of fluid is extremely small based on the volume of each nanochannel, which results in weak fluorescence signals. Prolonging the exposure time of the microscope is an effective way to enhance the signals; however, it also becomes a barrier to real-time fluorescent observation.

## Conclusions

In this paper, we present a new and reliable fabrication process based on nano-IndL to develop all-transparent polymer-based and encapsulated nanofluidic devices. The probe equipped in a nanoindenter is used to scratch size-controllable nanochannels on PMMA substrates, and then those nanostructures are transferred to PDMS substrates by performing the molding twice. By combining photolithography with soft lithography, PDMS ‘cap’ substrates with opposite microchannels and holes for the inlet/outlet connectors are also prepared. After alignment and bonding of the two PDMS pieces, all channels are sealed to achieve the transparent system for the study on nanofluidics. Both non-fluorescent and fluorescent liquid flow experiments are conducted to verify the functionality of the fabricated device and estimate the flow rates in the nanochannels. Although the precise alignment and fluorescent observation remain problems that need to be addressed, the device has great value for fundamental research on nanofluidics applications.
